# Prophage-Encoded Staphylococcal Enterotoxin A: Regulation of Production in *Staphylococcus aureus* Strains Representing Different *Sea* Regions

**DOI:** 10.3390/toxins7124889

**Published:** 2015-12-09

**Authors:** Nikoleta Zeaki, Yusak Budi Susilo, Anna Pregiel, Peter Rådström, Jenny Schelin

**Affiliations:** Applied Microbiology, Department of Chemistry, Lund University, Lund 22100, Sweden; nikoleta.zeaki@tmb.lth.se (N.Z.); yusak.budi_susilo@tmb.lth.se (Y.B.S.); annapregiel@gmail.com (A.P.); peter.radstrom@tmb.lth.se (P.R.)

**Keywords:** *Staphylococcus aureus*, enterotoxin A, prophage, *Siphoviridae*, staphylococcal food poisoning

## Abstract

The present study investigates the nature of the link between the staphylococcal enterotoxin A (SEA) gene and the lifecycle of *Siphoviridae* bacteriophages, including the origin of strain variation regarding SEA production after prophage induction. Five strains representing three different genetic lines of the *sea* region were studied under optimal and prophage-induced growth conditions and the *Siphoviridae* lifecycle was followed through the phage replicative form copies and transcripts of the lysogenic repressor, *cro*. The role of SOS response on prophage induction was addressed through *recA* transcription in a *recA*-disruption mutant. Prophage induction was found to increase the abundance of the phage replicative form, the *sea* gene copies and transcripts and enhance SEA production. Sequence analysis of the *sea* regions revealed that observed strain variances were related to strain capacity for prophage induction, rather than sequence differences in the *sea* region. The impact of SOS response activation on the phage lifecycle was demonstrated by the absence of phage replicative form copies in the *recA*-disruption mutant after prophage induction. From this study it emerges that all aspects of SEA-producing strain, the *Siphoviridae* phage and the food environment must be considered when evaluating SEA-related hazards.

## 1. Introduction

Staphylococcal food poisoning (SFP) is one of the most common food-borne intoxication diseases, caused by enterotoxins produced mainly by *Staphylococcus aureus* (*S. aureus*) strains. Staphylococcal enterotoxins have been the cause of 6.4% of food-borne outbreaks in the European Union (EU) in 2012, placing bacterial toxins as the third most common outbreak causative agent in the EU [1]. In the USA, *S. aureus* was ranked as one of the five most frequent causes of food-borne outbreaks with more than 240,000 illnesses annually [2]. This reflects how staphylococcal outbreaks are not only a public health problem, but also an economical challenge for the social health system in developed countries.

Until now, 22 staphylococcal enterotoxins (SEs) and enterotoxin-like proteins (SEls) are known [3]. They differ on the encoding genetic element (*i.e.*, plasmids, prophages, staphylococcal pathogenicity islands (SaPIs), vSa genomic islands or the staphylococcal cassette chromosome SCC), molecular weight (22–29 kDa), amino acid sequence or mode of action [3,4]. All SEs though, have been characterized with superantigenic activity, proteinase resistance and high stability in a wide range of pH and temperature [3,5,6]. The latter features make them extremely challenging for food safety, since they can persist even after food has been processed or cooked.

The enterotoxin involved in around 80% of SFP outbreaks is enterotoxin A (SEA) [3,5,7]. Unlike most other enterotoxins, SEA is not regulated by the accessory gene regulator (*agr*) or the staphylococcal accessory regulator (*sar*) and thus still poses a challenge regarding its regulatory mechanism. What is known is that the gene encoding for SEA, the *sea* gene, is located on the genome of *Siphoviridae* bacteriophages. These phages are temperate bacteriophages closely related to lambda (λ) and their life cycle is characterized by two phases, the lysogenic and the lytic phase [8]. During lysogeny the phage DNA is integrated into the bacterial chromosome and is steadily transferred through generations. In the lytic phase, the phage genome is excised from the chromosome, circularizes and replicates using the cell’s machinery. These dsDNA genome copies are often referred to as the replicative form (RF). Ultimately new phages will be formed that will lyse the bacterial cell [9,10].

Borst and Betley in 1994 [11] were the first to observe differences on the SEA levels produced by different *S. aureus* strains (high and low SEA-producing strains) and suggested an association with the *sea*-carrying prophage. Later, Wallin-Carlquist *et al.* [12] showed the existence of two *sea* variants, *sea_1_* and *sea_2_*, where high SEA-producing strains bore the *sea_1_* variant while *sea_2_* was found in low SEA producing strains. In 2012, Cao *et al.* [13] demonstrated that the life cycle of the *sea*-carrying phages influences the *sea* gene expression and the levels of SEA produced by *S. aureus* strains. It was also proved that some high SEA-producing strains had the ability to produce increased amounts of SEA when their cultures were subjected to prophage inducing conditions using mitomycin C (MMC). Accordingly, the high SEA-producing group was divided into two sub-groups, the inducible one, where higher SEA levels were observed and the non-inducible one, with no impact of induction on the SEA levels. A long *sea* transcript, as it was designated, was also detected and quantified. It was presumed to originate from a latent promoter (P_2_), located upstream the endogenous *sea* promoter (P_1_). This long transcript could only be detected in the inducible high SEA-producing strains.

It is known from studies on the λ-phage that the switch from lysogenic to lytic phase is controlled by the cell’s SOS response mechanism and specifically by the RecA activator protein [14]. When the SOS response is activated the RecA protein directly stimulates auto-proteolysis of the lytic repressor, *cI*, which in turn allows transcription of the anti-repressor, *cro*, that induces the lytic cycle of the phage [9,10,15], where transcription of relevant early and late phage lytic genes is initiated. The *sea* gene is located in the late gene region on the *Siphoviridae* phage genome; downstream the late lytic promoters, and therefore transition to the lytic phase could potentially activate and/or even enhance its transcription along with the phage’s late lytic genes.

The aim of the present study was to determine the role of the *Siphoviridae* phages on *sea* gene transcription and SEA production especially by identifying and characterizing the nature of the link between the phage life cycle and SEA produced. The impact of *S. aureus’* SOS response mechanism on the phage life cycle and SEA production was investigated. Five *S. aureus* strains representing the three different genetic lines of the *sea* region were grown and thoroughly investigated under optimal and MMC induced laboratory growth conditions. A *recA-*disruption mutant strain was constructed and studied under the same growth conditions. To understand how the behavior of the *sea*-carrying phages affects the SEA produced by different strains, the increase in phage RF copies was explicitly followed, to our knowledge, for the first time in *S. aureus* and correlated with that of the *sea* gene copies. The presence of *sea* transcripts under both control and MMC induced conditions was recorded and correlated time wise with the presence of RF in the cells and the amounts of SEA produced. By specifically monitoring RF the impact of the phage life cycle on *sea* transcription and SEA production was directly demonstrated. A significant (*p* < 0.05) increase in RF copy levels was detected under induced conditions, and this was further related to the activation of the SOS response mechanism of *S. aureus*.

## 2. Results

### 2.1. Inducible High SEA-Producing Strains

**Figure 1:**
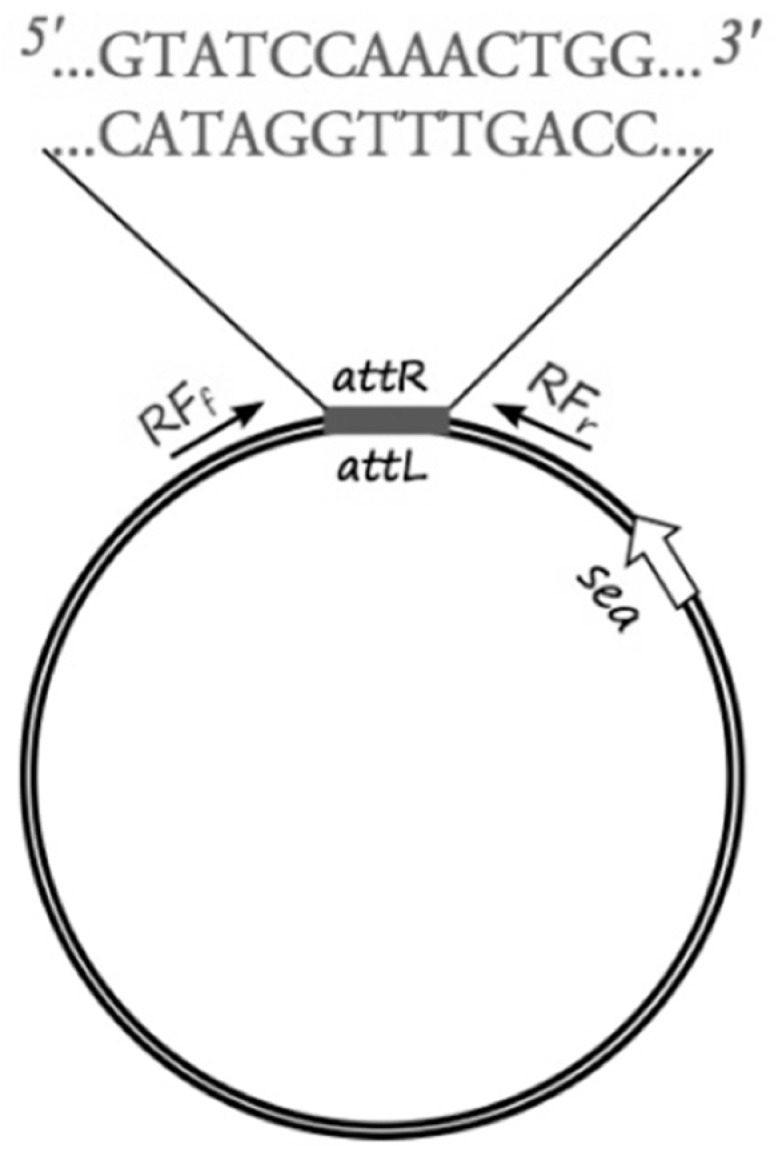
Schematic representation of the circular form of the *sea*-carrying *Siphoviridae* bacteriophage genome. The sites of integration to the bacterial chromosome are indicated as *attR* and *attL* and the nucleotide sequence of the respective cohesive ends is shown above. The primers designed for the specific detection and monitoring of the RF (circular form of the phage genome) are noted as RF_f_ and RF_r_. The *sea* gene is indicated by the white arrow.

To study the link between prophage induction and SEA production, the circular form of RF was explicitly quantified (Figure 1). 

**Figure 2:**
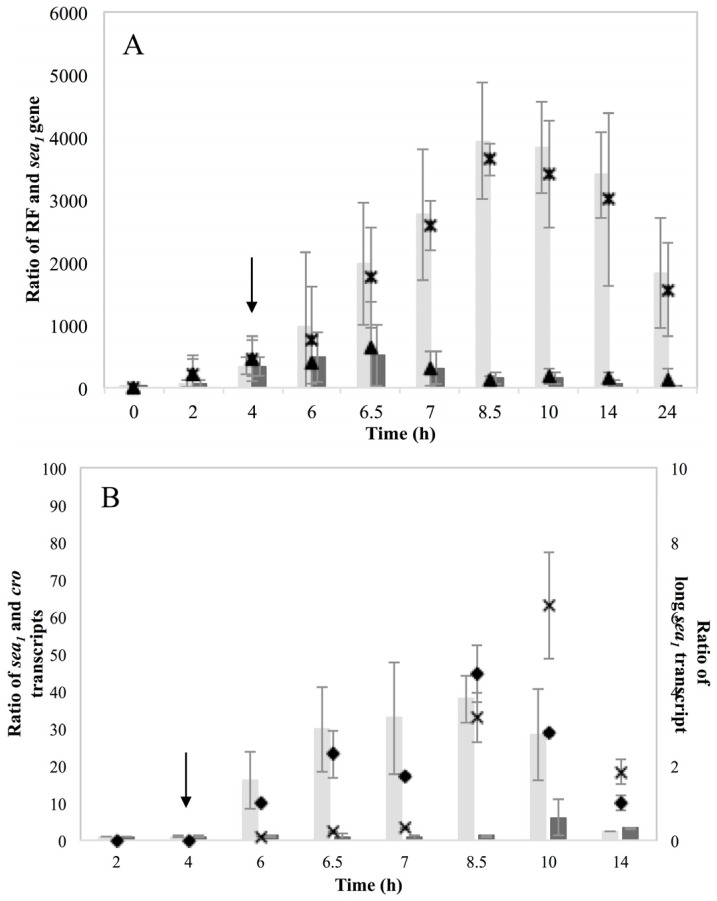
*Staphylococcus aureus* (*S. aureus*) strain Sa17 grown in Brain Heart Infusion (BHI) under control and Mitomycin C (MMC) induced conditions. Average values including standard deviations (^┬^, _┴_) of three independent experiments are presented. In the MMC induced culture, the point of induction at 4 h is indicated with an arrow. Time is represented in the *X*-axis in hours (h). (**A**) Ratio of RF and *sea* copy levels (*Y*-axis) as calculated based on Cq values and according to the equation ratio = (E _target_) ^ΔCq target(control-sample)^. Bars represent RF copy levels; dark grey bars designate RF levels for the control culture while light grey bars for the MMC induced culture. The *sea_1_* gene copy levels are represented by symbols. The levels for the control culture are designated the symbol (▲), while the levels for the MMC induced culture are designated the symbol (×); (**B**) Ratio (left *Y*-axis) of total *sea_1_* transcript represented in dark grey bars for the control culture and light grey bars for the MMC induced, and *cro* transcripts represented by the symbol (×). Ratio of long *sea_1_* transcript (right *Y*-axis) is designated the symbol (♦). The *cro* and long *sea_1_* transcripts were only detected in the MMC induced culture.

**Figure 3:**
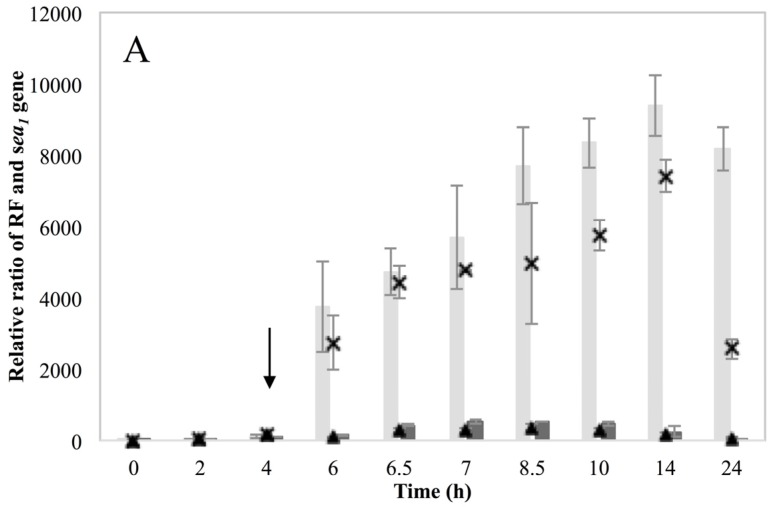

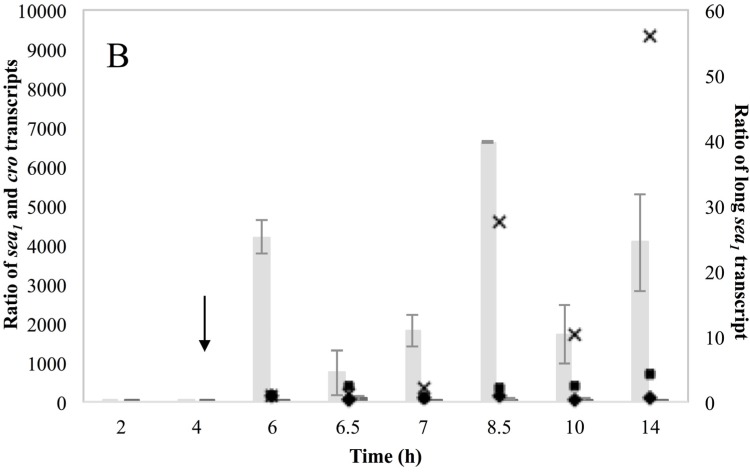
*S. aureus* strain Sa48 grown in BHI under control and MMC induced conditions. Average values including standard deviations (^┬^, _┴_) of three technical replicates are presented. In the MMC induced culture, the point of induction at 4 h is indicated with an arrow. Time is represented in the *X*-axis in hours (h). (**A**) Ratio of RF and *sea* copy levels (*Y*-axis) as calculated based on Cq values and according to the equation ratio = (E _target_) ^ΔCq target(control-sample)^. Bars represent RF copy levels; dark grey bars designate RF levels for the control culture while light grey bars for the MMC induced culture. The *sea_1_* gene copy levels are represented by symbols. The levels for the control culture are designated the symbol (▲), while the levels for the MMC induced culture are designated the symbol (×) (**B**). Ratio (left *Y*-axis) of total *sea_1_* transcript represented in dark grey bars for the control culture and light grey bars for the MMC induced, and *cro* transcripts represented by the symbol (■) for the control culture and (×) for the induced. Ratio of long *sea_1_* transcript (right *Y*-axis) is designated the symbol (♦). The long *sea_1_* transcript was only detected in the MMC induced culture.

The RF and *sea_1_* gene copy levels for the two inducible high SEA-producing strains Sa17 and Sa48 revealed a significant (*p* < 0.05) increase under MMC induced conditions (Figure 2A and Figure 3A). For Sa17, the RF levels between induced and control growth conditions exhibited on average a difference of 65 folds at the end of cultivation. Sa48 exhibited a more profound increase on the RF copy levels of the induced culture, which was in average 400 folds higher in ratio than the control culture at the end of incubation. Despite the different magnitude in the calculated ratios, the two strains followed a similar response to prophage induction with respect to RF and *sea_1_* gene copies. This pattern was further observed for growth, the presence of *sea* transcripts and SEA production. MMC induction caused a two log units reduction on the viable counts of both strains, compared to the control (Supplementary Figures S1a and S2a). Total *sea_1_* transcripts (originating from the endogenous P_1_ promoter) detected after MMC induction were found increased for both strains. Sa48, however, exhibited enhanced and prolonged transcription compared to Sa17, as shown by the presence of the detected transcripts (Figure 2B and Figure 3B). The long *sea_1_* transcript, originating from the P_2_ latent promoter, was detected in both strains exclusively under induced conditions. Transcripts of the lysogenic repressor *cro* were enhanced after MMC induction in both Sa17 and Sa48 strains. For Sa48, a basal *cro* transcription was also detected under control conditions. The impact of prophage induction in these strains was likewise evident for SEA production. Sa17 produced on average 2.5 times higher SEA levels in the induced culture compared to the control, while Sa48 exhibited a difference of almost seven times on average between the two conditions (Supplementary Figures S1b and S2b).

### 2.2. Non-Inducible High SEA-Producing Strains

**Figure 4:**
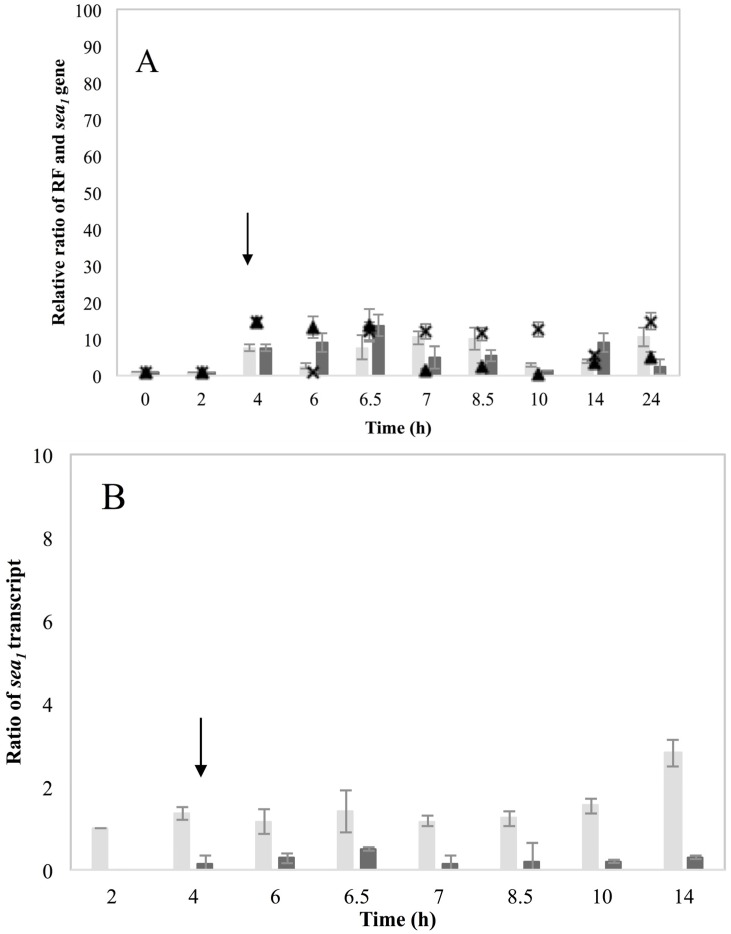
*S. aureus* strain, Sa21 grown in BHI under control and MMC induced conditions. Average values including standard deviations (^┬^, _┴_) of three independent experiments are presented. In MMC induced culture, the point of induction at 4 h is indicated with an arrow. Time is represented in the *X*-axis in hours (h). (**A**) Ratio of RF and *sea* copy levels (*Y*-axis) as calculated based on Cq values and according to the equation ratio = (E _target_) ^ΔCq target(control-sample)^. Bars represent RF copy levels; dark grey bars designate RF levels for the control culture while light grey bars for the MMC induced culture. The *sea_1_* gene copy levels are represented by symbols. The levels for the control culture are designated the symbol (▲), while the levels for the MMC induced culture are designated the symbol (×); (**B**) Ratio of total *sea_1_* transcript represented in dark grey bars for the control culture and light grey bars for the MMC induced. The *cro* and long *sea_1_* transcripts were not detected in either the control or MMC induced cultures.

The high but non-inducible SEA-producing strains, Sa21 and Mu50, were investigated to pinpoint differences compared to the high inducible strains. The RF and *sea_1_* gene copy levels of Sa21 were distinctly lower than the inducible strains. No noteworthy difference in RF levels was found between induced and control growth conditions in this strain and *sea_1_* gene copy levels remained below 20 in ratio (Figure 4A). As for the inducible strains, Sa21 growth, *sea* transcription and SEA production followed the response observed for the RF and *sea_1_* gene. MMC induction had no evident impact on the viable counts of Sa21 (Supplementary Figure S3a). The total *sea_1_* transcript was detected throughout the experiment, though in very low levels. A moderate difference on the transcript levels present was observed between the induced and control samples however not as pronounced as in the case of the inducible strains (Figure 4B). SEA levels produced under the two growth conditions were rather similar, and remained overall below 3000 ng·mL^−1^ for both cultures (Supplementary Figure S3b). The long *sea_1_* and *cro* transcripts were not detected at any condition and time point.

**Figure 5:**
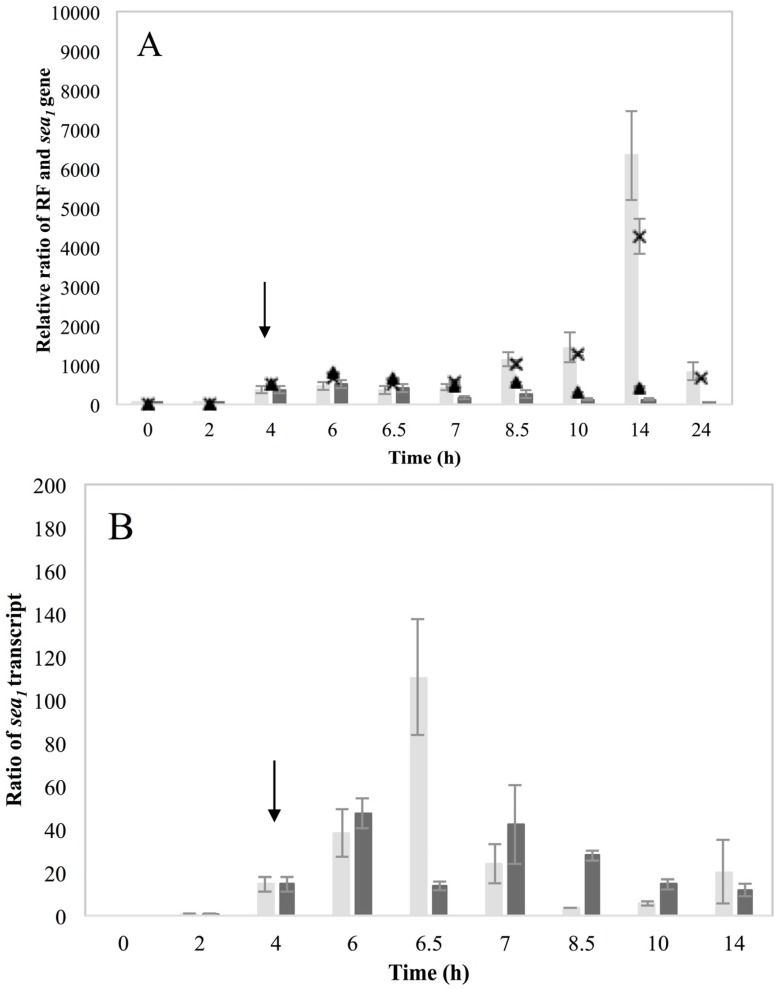
*S. aureus* strain Mu50 grown in BHI under control and MMC induced conditions. Average values including standard deviations (^┬^, _┴_) of three technical replicates are presented. In MMC induced culture the point of induction at 4 h is indicated with an arrow. Time is represented in the *X*-axis in hours (h). (**A**) Ratio of RF and *sea* copy levels (*Y*-axis) as calculated based on Cq values and according to the equation ratio = (E _target_) ^ΔCq target(control-sample)^. Bars represent RF copy levels; dark grey bars designate RF levels for the control culture while light grey bars for the MMC induced culture. The *sea_1_* gene copy levels are represented by symbols. The levels for the control culture are designated the symbol (▲), while the levels for the MMC induced culture are designated the symbol (×); (**B**) Ratio of total *sea_1_* transcript represented in dark grey bars for the control culture and light grey bars for the MMC induced. The *cro* and long *sea_1_* transcripts were not detected in either the control or MMC induced cultures.

Interestingly, the non-inducible strain Mu50 exhibited a marked increase in RF levels and *sea_1_* gene copies 10 h after MMC induction (14 h time point) (Figure 5A). The impact of prophage induction was also evident on growth of this strain, as a 2 log unit reduction was observed between 6 and 7 h of incubation under induced conditions (Supplementary Figure S4a). The detected total *sea_1_* transcripts were markedly higher (about eight folds) in the induced culture at 6.5 h of growth compared to those detected in the control (Figure 5B). This response to prophage induction was however not observed neither for the long *sea_1_* nor the *cro* transcript, as detection of both was not achieved. The SEA levels formed by Mu50 gave yet again an interesting observation, as the control culture produced up to twice as much SEA as the induced culture (Supplementary Figure S4b).

### 2.3. Low SEA-Producing Strain, Sa51

A strain, Sa51, previously characterized as low SEA-producing was investigated regarding the life cycle of its *sea_2_*-carrying prophage. From previous studies, this strain, exhibited very low SEA levels and no expression of total or long *sea_2_* transcript was detected in the different growth conditions investigated [13]. The present results on total *sea_2_*, long *sea_2_*, and *cro* transcripts as well as SEA produced, conform to the previous findings for Sa51. Surprisingly, new findings regarding the RF and *sea_2_* gene copy levels of Sa51 after MMC induction were discovered in this study. Specifically, increased levels of RF and *sea*_2_ gene copies were observed after 10 hours of growth under induced conditions even though growth of Sa51 was not found affected (Supplementary Figure S5).

### 2.4. Sequence Analysis of *sea* Gene Region

DNA sequence analysis of 11 *S. aureus* strains carrying the *sea* gene was performed to localize any existing differences within a 3.6 kb gene region including the *sea* gene (Table 1).

**Table 1:** *S. aureus* strains used for analysis of the 3.6 kb gene region including the *sea* gene.

Strain Name	NCBI ^a^ Accession Number	SEA ^b^ Production ^c^	Inducible
**MW2**	NC_003923.1	High	Yes
**Mu50**	NC_002758.2	High	No
**MRSA252**	NC_002952.2	Low	No
**Newman**	NC_009641.1	Low	No
**Sa17**	KP402066	High	Yes
**Sa21**	KP402067	High	No
**Sa45**	KP402068	High	Yes
**Sa48**	KP402069	High	Yes
**Sa51**	KP402070	Low	No
**Sa53**	KP402071	High	Yes
**Sa54**	KP402072	Low	No

**^a^** National Center for Biotechnology Information; **^b^** Staphylococcal enterotoxin A; **^c^** Cao, Zeaki, Wallin-Carlquist, Skandamis, Schelin and Rådström [13].

**Figure 6:**
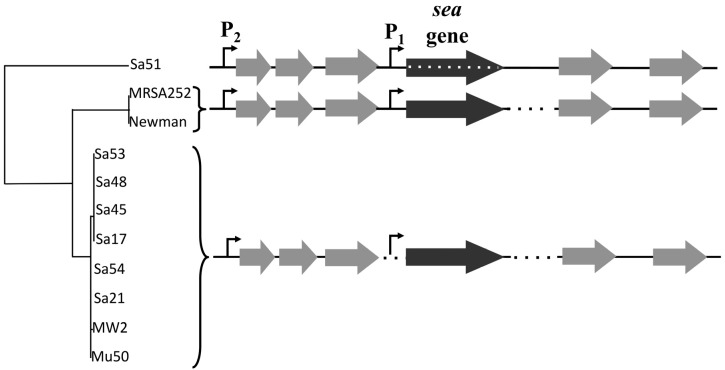
The *sea* gene region (3.6 kb long) of 11 *S. aureus* strains. The black arrow represents the *sea* gene while the grey arrows represent genes in the surrounding regions. The endogenous *sea* promoter is marked as P_1_ and the latent promoter as P_2_. Observed sequence differences are represented with dotted lines.

The aim was to understand whether the strain-dependent SEA production could be attributed to particular sequence differences. The outcome was compiled in a tree showing three distinct strain branches (Figure 6). Interestingly, the inducible and non-inducible high SEA-producing strains carrying the *sea_1_* gene belonged to the same branch and no key sequence differences were detected that could explain the different capacity for prophage induction. Another branch was formed by two fully sequenced strains, Newman and MRSA252, which carry the *sea_2_* gene and produce intermediate levels of SEA as found by Cao *et al.* [13]. The third branch was composed of only Sa51 that intriguingly was found distinctly different compared to the high SEA-producing strains. Several additional stretches of nucleotides were detected both around the P_1_ promoter and downstream of the *sea* gene along with 119 point mutations inside the actual *sea* gene for this strain (Supplementary Figure S6a,b). These mutations resulted in an altered amino acid sequence where 59 amino acids, including the start codon (Met to Ile), out of a total of 257, differed compared to the high SEA producing strains (Supplementary Figure S6c). To investigate the impact of amino acid differences on SEA detection specificity, ELISA was performed with antibodies for SEA and SEE. These two toxins exhibit the highest degree of resemblance compared to the rest of the staphylococcal enterotoxins (81.7%) [16]. The obtained results for both SEA and SEE were less than 10 ng·mL^−1^ throughout the incubation period (data not shown).

### 2.5. Sa17 recA-Disruption Mutant Strain

**Figure 7:**
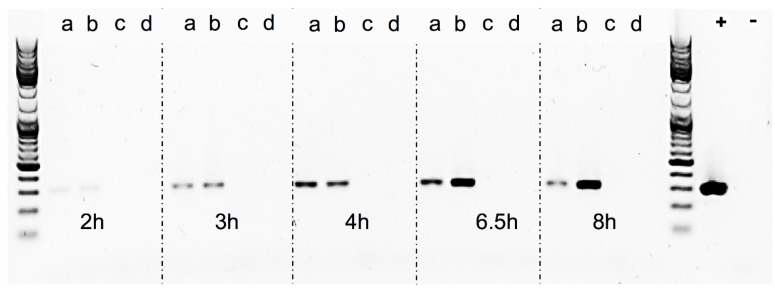
Detection of RF by PCR and agarose gel electrophoresis in *S. aureus* strains Sa17 wild type and Sa17 *recA*-disruption mutant grown in BHI under induced and controlled conditions: (**a**) control Sa17 wild type; (**b**) induced Sa17 wild type; (**c**) control Sa17 *recA*-disruption mutant; and (**d**) induced Sa17 *recA*-disruption mutant. Positive and negative control are denoted + and −, respectively. DNA ladder mix is included to verify the RF product size.

To explore the impact of *S. aureus’* SOS response mechanism on the phage life cycle and SEA production the *recA* gene in the inducible strain Sa17 was disrupted and Sa17 wild type and *recA*-disruption mutant, were grown under control and induced conditions. Sa17 *recA*-disruption mutant exhibited a similar growth pattern to Sa17 wild type under both growth conditions (data not shown). The impact on prophage induction was assessed by the amount of RF specific amplicon formed as measured from band intensity in gel electrophoresis (Figure 7 and Supplementary Table S2). In Sa17 wild type, induced culture amplicons of RF were observed with a stronger intensity compared to the control culture. On the contrary, Sa17 *recA*-disruption mutant was characterized by the absence of RF amplicons for both induced and control growth conditions. Investigation of the SEA levels produced in both induced and control cultures of the Sa17 *recA*-disruption mutant further showed a noticeable difference where the control exhibited five times higher SEA levels than the induced culture (Supplementary Figure S7).

## 3. Discussion

The role of the *sea*-carrying phages’ life cycle and impact of prophage induction on the regulation of *sea* gene transcription, has been discussed in previous studies of Sumby and Waldor [17] and Cao *et al.* [13] where a phage activated long *sea* transcript was linked with increased SEA production. However, the transition from lysogeny to the lytic mode in the life cycle of temperate bacteriophages brings about changes both on the transcription pattern of phage-associated genes, and their copy numbers available for potential transcription, through the increase of RF levels.

Investigation of RF levels in the present study concluded that the inducible high SEA-producing strains (Sa17 and Sa48), exhibited marked increase in the levels of RF copies after prophage induction, in a pattern that corresponded to the increase in the levels of *sea_1_* gene copies of the induced cells. This observation demonstrates the impact of prophage induction on *S. aureus* virulence, as excision and replication of the phage genome increases the *sea* gene copies in the cells. Detection of the long *sea_1_* transcript and the increase of total *sea_1_* transcripts noted, further shows the series of events leading to high SEA production by Sa17 and Sa48, after prophage induction. The observed similar transcriptional behaviour of *cro* and the long *sea_1_* transcript indicates similar regulation of their expression and supports the conception of the phage activated P_2_ promoter. The lysogenic repressor is known, from studies on phage λ, to increase in levels under prophage induction, while during lysogeny only basal transcription of *cro* occurs [10,15]. The different levels of SEA observed between Sa17 and Sa48, were justified by the higher levels of RF, *sea_1_* gene copies and total *sea_1_* transcript in Sa48. Bearing in mind that the type of phages carrying the *sea* gene are characterized by superinfection immunity [10], this behavior could be attributed to enhanced phage replication and prolonged mRNA transcription or stability in Sa48.

These new findings enlightening the link in which prophage induction affects SEA production, were also indirectly supported by the behavior of the non-inducible Sa21 strain. This strain behaved as if the changes in the environment by the addition of MMC were either not sensed by the cell or that the phage was unable to excise and replicate after the signal of environmental change was received. The dearth of transcripts from the latent *sea* promoter, P_2_, and the lysogenic repressor, *cro*, favor the first hypothesis. Either way, the different behavior of Sa21 towards prophage induction could be due to key differences in the phage genome sequence, to differences in regulatory proteins of the SOS response system or to a combination of both. Equally intriguing was the equivocal behavior of the non-inducible strain Mu50. This strain has also been previously found to respond similarly towards prophage induction as Wallin-Carlquist *et al.* [12] showed that acetic acid increased Mu50 *sea* gene copy numbers at pH 5.5 but the expected boost on SEA levels was not observed. In regard to what has been observed so far for the different SEA-producing strains, it can be deduced that Mu50 could behave as an inducible high SEA-producer, when considering only the ability for prophage induction; however, possible impaired transcription efficiency could obstruct the transcription of the *sea* gene. Likewise, the observation on the induction capacity of the low SEA-producer Sa51, which was only apparent on the RF and *sea_2_* gene copy levels, but not on *sea_2_* transcripts or the SEA produced, further relates to the issue of transcription efficiency in some strains and the role that sequence differences could have on the ability of the phage to excise and replicate.

The analysis of the 3.6 kb gene region including the *sea* gene complemented previous data by Wallin-Carlquist *et al.* [12], where a map for the *sea_1_* and *sea_2_* gene alleles had been created. The lack of key sequence differences in this region between the inducible and non-inducible high SEA-producers further supports the critical role of the different *sea*-carrying phages on *S. aureus* virulence. The possibility of sequence differences outside the region analyzed in this study, could, however, explain the capacity of the phages to excise and replicate, and thus needs further investigation. The differences observed for Sa51 could possibly explain its response to prophage induction, as the mutations in the *sea* gene region alter not only the SEA amino acid sequence but could also affect the transcription and translation process in this strain. Interestingly, Soltis *et al.* [18] had also reported a gene named as *sezA* that bore differences from the previously identified *sea* gene. This *sea* variant, though transcribed into stable mRNA could not be translated into a SEl protein, as it was deficient of an appropriate translation initiation codon. Specifically, the *sezA* gene differed at the start codon by a single nucleotide deletion. Further investigation of the *sea_2_* gene will elucidate the present observations.

Altogether, the findings presented in this study, lead to the realization that the phage life cycle affects SEA production in two different ways. Initially, activation of the phage with consequent replication of its genome increases the pool of the *sea* gene copies in the cell and thus the possibility to produce more *sea* mRNA. This gene dose effect is one of the reasons behind the observed increase in SEA production, which is noted for the inducible high SEA-producing strains but not for the non-inducible one. Furthermore, as stated above, the *sea* gene is located in the late lytic region of the phage genome. Activation of the lytic promoters could accordingly have an impact on the level of *sea* transcription. Detection of the long *sea_1_* transcript only for the inducible strains and during induced conditions is a strong indication that the putative latent promoter P_2_ is regulated by the phage and specifically activated when it is entering the lytic phase generating transcripts including *sea*. The demonstration that prophage induction is linked with the activation of the RecA protein highlights the critical role of environmental changes on phage activation and thus the risk for SFP.

## 4. Experimental Section

### 4.1. Bacterial Strains and Growth Conditions

Four food isolates of *S. aureus*, Sa17, Sa21, Sa48, and Sa51 (donated from SP Food and Bioscience, the former Swedish Institute for Food and Biotechnology, SIK, Göteborg, Sweden), and a fully sequenced strain Mu50 (LGC Promochem, London, UK), were studied. Brain Heart Infusion (BHI) broth (Difco Laboratories, BD Diagnostic Systems, Le Point de Claix, France), was used as growth medium throughout the study. Prior to inoculation, each strain was streaked on BHI agar plates (Difco Laboratories, BD Diagnostic Systems, Le Point de Claix, France), from −80 °C glycerol stocks, and incubated for 16 to 18 h (overnight) at 37 °C. A single colony of each strain was selected and sterilely transferred to a 50 mL falcon tube containing 25 mL of BHI broth. The pre-cultures were grown overnight in a rotating incubator (New Brunswick Scientific, Innova 40/40R incubator Shakers, Eppendorf AG, Hamburg, Germany) at 37 °C and 200 rpm shaking. To remove secreted metabolites and enterotoxins produced by *S. aureus* during overnight growth, pre-cultures were centrifuged (3220× *g*, 10 min, 4 °C, 5810R table centrifuge, Eppendorf AG, Hamburg, Germany) and cell pellets were washed twice in 25 mL of 0.9% sterile NaCl solution (Merck Millipore, Darmstadt, Germany) and re-suspended by vortexing in the same volume of 0.9% sterile NaCl solution. Optical density (OD) of the washed pre-cultures was measured at 620 nm (UV/Visible Spectrophotometer Ultrospec 2100 pro, GE Healthcare, Little Chalfont, UK) and the adequate inoculum volume for a starting OD of 0.1 in 400 mL BHI (1 L flask) was calculated. Thereafter, cultures were grown under optimal conditions (37 °C, 200 rpm) for about 4 h until OD ≈ 5 (mid-exponential growth phase). At this point, they were sterilely divided into half volumes and transferred to new flasks of 500 mL size. One half was then induced using mitomycin C (MMC) (Duchefa Biochemie, Haarlem, The Netherlands) in a concentration of 1 mg·mL^−1^ of culture, while the other was kept as a control (optimal conditions). Both cultures were then further incubated at 37 °C, 200 rpm. Samples were collected at 0 h, 2 h, 4 h, 6 h, 6.5 h, 7 h, 8.5 h, 10 h, 14 h and 24 h for OD, CFU (colony forming units), DNA and RNA isolation and ELISA analysis. For strains Sa17 and Sa21 three independent experiments were performed, while strains Sa48, Sa51 and Mu50 were investigated once. CFU determination was performed by serially diluting 1 mL of sample into 9 mL of 0.9% sterile NaCl solution. A volume of 100 μL of the appropriate dilutions was then plated, in duplicates, on BHI agar plates. The plates were incubated approximately 24 h at 37 °C before enumeration of colonies. Results were calculated and expressed as log cfu·mL^−1^.

### 4.2. DNA Extraction

Two methods, genomic and plasmid DNA extraction were used in this study to evaluate the quantity of extracted RF and the quality of the subsequent qPCR analysis. Prior to extraction, cells were washed once with sterile 0.9% NaCl solution. Purification of plasmid DNA was performed using the GeneJET Plasmid Miniprep Kit (Thermo Fisher Scientific, Waltham, MA, USA), as described in the producer’s protocol. Genomic DNA was extracted with the GeneJET Genomic DNA Purification Kit (Thermo Fisher Scientific, Waltham, MA, USA) according to the producer’s protocol for Gram-positive bacteria, including one modification of the lysis buffer. Specifically, lysis buffer was prepared using 5% lysozyme and 1% lysostaphine for more efficient extraction. Concentration of extracted DNA (ng·µL^−1^) and purity (A_260/280_) were measured using BioDrop spectrophotometer (BioDrop TOUCH UV/Visible Spectrophotometer, Integrated Scientific Solutions Inc., Walnut Creek, CA, USA). All DNA samples were stored at −20 °C until further analysis. Samples from both extraction methods were analyzed in qPCR and compared regarding the levels of the extracted RF. Both methods were equally successful in the isolation of RF and thus, plasmid extraction was chosen to continue further as it was the fastest isolation method.

### 4.3. RF Assay

The sequenced phage ΦSa3mw of *S. aureus* MW2 (Ac. No. BA000033.2) was used to pinpoint the region of the attachment sites of the prophage and bacterial chromosome ligation. Specific primers for sequencing (RF sequencing primers, Table 2) were designed, as shown in Figure 1, to amplify the region around the cohesive ends of the phage, only when in the circular form, in order to determine the exact sequence of the cohesive ends of six *S. aureus* strains (Sa17, Sa21, Sa48, Sa51, Sa54 and Mu50). PCR was conducted with Gene Amp 9700 thermal cycler (Perkin-Elmer, Waltham, MA, USA) using the proof-reading enzyme *Pfu* DNA polymerase (Thermo Fisher Scientific, Waltham, MA, USA). The following PCR protocol was used: initial denaturation at 95 °C for 1 min, followed by 30 cycles of denaturation at 95 °C for 30 s, annealing at 54 °C for 30 s and elongation at 72 °C for 3 min. A final extension step was added at 72 °C for 5 min and hold at 4 °C. Obtained PCR products were purified using GeneJET PCR Purification Kit from Thermo Fisher Scientific, Waltham, MA, USA. DNA concentration was measured using BioDrop spectrophotometer and diluted to a concentration of 10 ng·μL^−1^. 15 μL of each PCR product and 10 μL of 10 μM of each primer (forward, reverse) were sent for sequencing at Eurofins MWG GmbH, Germany. The individual sequence reads were assembled and confirmed by the overlapping regions. Manual quality check and assembly of the sequencing results were performed using Serial Cloner 2.6.1 (Franck Perez, Serial Basics 2004-2013, http://serialbasics.free.fr/Serial_Cloner.html). Analyses of the obtained sequences demonstrated a common 13-nucleotide region among four out of the five investigated strains (no sequence data obtained for Sa21) that functioned as cohesive ends for the respective phages (Figure 1, Supplementary Table S1). The 13-nucleotide sequence region was aligned and verified through BLAST with the existing fully sequenced strains.

The sequence region surrounding and including the 13-nucleotide cohesive ends was used to create a specific qPCR assay that would allow the quantification of explicitly circular RF copies. Specific primers were designed, as shown in Figure 1, to amplify a 200-nucleotide long target with specific probes designed on the cohesive ends part of the target sequence.

**Table 2:** Sequences of primers and probes with fluorescent dyes used for qPCR analyses of *sea* gene, the replicative form of the bacteriophage genome (RF) and the sequencing of RF cohesive ends.

Target	Primer/Probe	Sequence (5′ T 3′)
***sea_1_ gene***	ESA-1	5-ATGAGTTGGGCAAGATGGTT-3
	Tox A reverse	5-GGACTTGTTGTCCACGTTAGG-3
	Tox A-Fluo 1	CCTTTGGAAACGGTTAAAACGAATAAGAA-FL
	Tox A-Red 1	LC-R640-TGTAACTGTTCAGGAGTTGGATCTTCA p
***sea_2_ gene***	*sea_2_* forward	CAATATATACAAAGGGAAAAAAGTG
	*sea_2_* reverse	CAGAAGAAGGGTGAAACTCA
	*sea_2_* FL	ATCCATAAGTTAATCGGTACTTTCTTTTC-FL
	*sea_2_* LC	640-TCCTCCAATTGATTATTATCATGTAACGT p
**RF assay**	forward	AAAATATAGCAATAACTACATCCG
	reverse	AAGTCCCTAAAAAGTCCCTA
	FL	ATGTTAAAAGTCTCCAGTTTGGATACA-FL
	LC	640-AGAAACCTTGTAACAACAGTATTTATTGGG p
**Long transcript**	forward	ATGAGTTGGGCAAGATGGTT
	reverse	GGACTTGTTGTCCACGTTAGG
	TaqMan BBQ	FAM-CATACTGCAAGTGAAGTTGGGAAGTGT-BBQ
**RF sequencing primers**	forward	CGCACGCATTAAGACACACT
	reverse	ATGCCCCATAAACAACCCTT
***cro* gene**	*cro* forward	TTAGCAATTTTTAAAGCACG
	*cro* reverse	GCAATAAGTGCAAGAGAGTTATAT
	*cro* FL	GCTTTCTCTACTTGGATGAAATACTCTCT-FL
	*cro* LC	LC640-AAATCAAAACCTTTTTCTGTACCTGACA-PH

### 4.4. RNA Extraction

A modified version of the protocol described by Lövenklev *et al.* 2004 [19] was used for RNA extraction. In brief, cells from 5 mL culture were harvested at 4 °C, 3220× *g*. The resulting pellet was immediately frozen in liquid nitrogen and stored at −80 °C until extraction of RNA. For RNA extraction, each sample pellet was resuspended in 500 μL ice cold TES buffer, pH 7.5 (50 mM Tris (BDH Prolabo, VWR International, Stockholm, Sweden), 5 mM EDTA and 50 mM NaCl (Merck Millipore, Germany)) and the suspension was transferred to Precelly lysing kit tubes (VK 0.1) (Bertin technologies, Montigny-le-Bretonneux, France). Cells were disrupted in a Precellys 24 unit, in a three cycle run of 60 s at 6500 rpm. RNA was isolated using two steps of phenol-chloroform (600 μL:100 μL) extraction followed by one chloroform (600 μL) purification step. Precipitation of RNA was achieved in 0.1 vol of 3 M NaAc (sodium acetate) (pH 4.8) and 2.5 vol of 95% EtOH for at least for 1 h at −80 °C and further washed with 600 μL of 70% EtOH (ethanol). The remaining traces of EtOH after expiration were removed by evaporation in room temperature, before the RNA was dissolved in 100 μL RNA storage solution (Ambion, Thermo Fisher Scientific, Waltham, MA, USA). Concentration of extracted RNA (ng·μL^−1^) and purity (A_260/280_) were measured using BioDrop spectrophotometer. Prior to transformation into cDNA by reverse transcription, all RNA samples were treated with DNase to degrade contaminating traces of DNA. For this reaction, 15 μL of each RNA sample was mixed with 15 μL autoclaved diethyl pyrocarbonate (Fluka Analytical, Sigma-Aldrich, Buchs SG, Switzerland)-treated water, 15U RQ1 RNase-free DNase (Promega Co., Madison, WI, USA) and 1× reaction buffer. The suspension was incubated at 37 °C for 45 min. Stop solution, in combination with incubation at 65 °C for 10 min, was further added to inactivate the DNase. All samples were stored at −80 °C until further analysis.

### 4.5. cDNA Synthesis

First-strand cDNA was synthesized in two separate reverse-transcription reactions using reverse primers specific to the total *sea*, long *sea* and *cro* transcripts (Table 2). The reactions were performed with a Gene Amp 9700 thermal cycler (Perkin-Elmer Cetus, Norwalk, CT, USA). The total volume of the mixture was 20 μL and contained 0.5 μg of total RNA, the reverse primer for each target at a concentration of 0.5 mM, each deoxynucleoside triphosphate (dATP, dTTP, dCTP, and dGTP; Roche Diagnostics GmbH, Penzberg, Germany) at a concentration of 5 mM, 20 U of RNasin RNase inhibitor (Promega Co., Madison, WI, USA), 5 mM dithiothreitol, 1× first-strand buffer, and 200 U of Superscript II RNase reverse transcriptase (Invitrogen, Thermo Fisher Scientific, Waltham, MA, USA). Autoclaved ddH_2_O was used both in the reaction mixture and to dilute mRNA samples. Before RT enzyme was added, the reaction mixture was heated to 65 °C for 5 min and then chilled on ice. After a brief centrifugation and addition of the RT enzymes, the reaction mixture was incubated at 42 °C for 50 min, and the reaction was terminated by incubation at 70 °C for 15 min. The cDNA was diluted 10-fold in autoclaved ddH_2_O before qPCR analysis. Transcript analysis was performed on samples until 14 h of growth, as it was shown before that transcription decreases notably at late growth phases [12,13].

### 4.6. Quantification of RF, *sea* Gene Copies, *sea* and *cro* Transcripts

To follow the levels of the bacteriophage RF and the *sea* gene, qPCR analysis of the purified plasmid samples was performed. To investigate the presence of the *sea* and *cro* transcripts, the cDNA synthesized from the extracted RNA was used for qPCR analysis. Specifically, a 20 μL qPCR reaction, including 10 ng of plasmid DNA template, was performed in a LightCycler 2.0 Carousel-Based System^®^ (Roche Diagnostics GmbH, Penzberg, Germany), using hybridization probes. The PCR mixture consisted of 1 PCR *Tth* buffer, 3.25 mM MgCl_2_, 0.2 mM of dNTPs mixture, 0.5 μM of both forward and reverse primers, 0.03 μM of each hybridization probe and 0.05 U of *Tth* polymerase. All reagents mentioned above were purchased from Roche Diagnostics GmbH, Germany, apart from probes and primers, which were supplied by TIB Molbiol GmbH, Berlin, Germany (listed in Table 2). Autoclaved ddH_2_O was used for all preparations and as negative control. The PCR protocol consisted of initial denaturation at 95 °C for 1 min, followed by 45 cycles of denaturation at 95 °C for 0 s (*i.e*., no hold at 95 °C), primer annealing at 46 °C for *sea_1_*, 49 °C for *sea_2_*, 50 °C for *cro* and 49 °C for RF, for 5 s, and extension at 72 °C for 25 s. A single fluorescence measurement was made at the end of the extension step. Cq values obtained were accepted when efficiency of amplification was between 1.8 and 2.0. Results are presented as calculated ratios between the reference sample (0 h sample; denoted “control” in the equation) and the target sample according to the equation of Pfaffl (ratio = (E _target_) ^ΔCq target(control-sample)^ [20]).

### 4.7. ELISA

ELISA for SEA and SEE was performed according to the revised laboratory protocol for staphylococcal enterotoxin A, described before by Wallin-Carlquist *et al.* [12]. Absorbance was measured at 405 nm with Multiskan Ascent^®^ spectrophotometer (Electron Corporation, Thermo Fisher Scientific, Waltham, MA, USA). Obtained absorbance values were plotted against toxin concentrations. Absorbance values for the standard samples were plotted against the known concentrations of SEA and SEE and standard curves were created. The concentrations of the unknown samples were calculated using the linear regression and expressed in ng·mL^−1^ of toxin.

### 4.8. Sequencing and Analysis of *sea* Gene Region

A 3.6 kb region including the *sea* gene and its 2 putative promoters was sequenced in 11 different isolates of *S. aureus* strains (Table 1). Sequencing primers for the food isolates (Sa17, Sa21, Sa45, Sa48, Sa51, Sa53, Sa54) were designed by multiple alignment of known sequences of *S. aureus* strains Mu50, MW2, Newman and MRSA252 from NCBI database with MUSCLE [21] and the primers were designed to have a 0.5 kb distance between them to support assembly and confirmation processes by overlapping regions. PCR was conducted with Gene Amp 9700 thermal cycler (Perkin-Elmer Cetus, Norwalk, CT, USA) using the proofreading enzyme Phusion DNA polymerase (Thermo Fisher Scientific, Waltham, MA, USA) and 1X Phusion GC Buffer. For strains MRSA252 and Newman the primer set YBSEA_9 and YBSEA_19 was used for amplification while the rest of the strains were amplified with the primer set of YBSEA_9 and YBSEA_18 (Table 3). The following PCR protocol was used: initial denaturation at 98 °C for 1 min, followed by 30 cycles of denaturation at 98 °C for 10 s, annealing at 47 °C and 55 °C, respectively, for 30 s and elongation at 72 °C for 3 min. A final extension step was added at 72 °C for 7 min and held at 4 °C. Obtained PCR products were purified using GeneJET PCR Purification Kit. DNA concentration was measured and diluted to a concentration of 10 ng·μL^−1^. Fifteen microliters of each PCR product was premixed with 2 μL of 10 μM of appropriate primer set and sequenced by Eurofins Genomics, Germany. Each individual sequence reads were assembled and confirmed by the overlapping regions. Manual quality check and assembly of the sequencing results were performed using Serial Cloner 2.6.1 and FinchTV 1.5.0 (Geospiza Inc., Seattle, WA, USA) followed by multiple sequence alignment of all obtained sequences using MUSCLE [21] and phylogenetic tree construction using TreeDyn [22].

**Table 3:** Primer sequences used for sequencing the 3.6 kb gene region including the *sea* gene and for the construction of the *recA*-disruption mutant.

Primer	Direction	Sequence	Target DNA	Target Strains
**YBSEA_1**	Forward	AAGTGGTAAAAAGTTATCTG	1.5–1 kb upstream *sea*	MW2, Sa17, Sa21, Sa45, Sa54
**YBSEA_2**	Forward	GTAGGCAGTTTTACAACGTC	1–0.5 kb upstream *sea*	MW2, Sa17, Sa21, Sa45, Sa54, Sa48, Sa53
**YBSEA_3**	Forward	TAAATGGCTCGTAGGTGCCA	0.5 kb upstream—start of *sea*	MW2, Sa17, Sa21, Sa45, Sa54, Sa48, Sa53
**YBSEA_4**	Forward	ATGAAAAAAACAGCATTTA	Start of SEA—0.5 kb inside *sea*	MW2, Sa17, Sa21, Sa45, Sa54, MRSA252, Sa48, Sa53
**YBSEA_5**	Forward	GTTAAAACGAATAAGAAAAA	0.5 kb inside SEA—0.2 kb downstream *sea*	All strains
**YBSEA_6**	Forward	ATTCCTTATTGCATTGATAG	0.2–0.7 kb downstream *sea*	All strains
**YBSEA_7**	Forward	TATGAAGAACCAAGAAGAAA	0.7–1.2 kb downstream *sea*	All strains
**YBSEA_8**	Forward	CAAGTAAAATAACAGTTGGA	1.2–1.5 kb downstream *sea*	All strains
**YBSEA_9**	Reverse	AATCAATCTCTCATGCCATA	1.5–1 kb downstream *sea*	All strains
**YBSEA_18**	Forward	TCAAACGCTGATAGTGCATA	2–1.5 kb upstream *sea*	MW2, Sa17, Sa21, Sa45, Sa54
**YBSEA_19**	Forward	CTGATGAGAACTATGATTAC	2–1.5 kb upstream *sea*	Newman, Sa51, MRSA252
**YBSEA_20**	Forward	GGTTTGATTGCTGAAGAGGT	1.5–1 kb upstream *sea*	Newman, Sa51, MRSA252
**YBSEA_21**	Forward	AAATCTGAAGAAAACGCTAA	1–0.5 kb upstream *sea*	Newman, Sa51, MRSA252
**YBSEA_22**	Forward	TGCAGTCATTACTGCATCAA	0.5 kb upstream—start of *sea*	Newman, Sa51, MRSA252
**YBSEA_101**	Forward	GGAGAAATNGAAGGTATNG	2–1.5 kb upstream *sea*	Sa48
**YBSEA_105**	Reverse	TATCTAGTGGCTGAAGTAGC	1.5–1 kb downstream *sea*	Sa53
**YB RecA-108**	Forward	AGTCAGTCGAGCTCTATGGAGAAATCTTTCGGTA		
**YB RecA-109**	Reverse	GTCAGTCGCGGCCGCAGTCTCTGGATTACCGAACA		
**YB RecA-110**	Forward	CCTTGTATAGTATTGGTAAGATA		
**YB RecA-111**	Reverse	GTATCGATAAGCTTGATATC		

### 4.9. Construction of recA Disruption Mutant

To create the *S. aureus* Sa17 *recA* disruption mutant, recombination technique with the plasmid pIMAY containing a chloramphenicol resistance gene was used [23]. A single *recA* fragment was amplified from *S. aureus* Sa17 strain with primers YBRecA-108 and YBRecA-109 (Table 3) and inserted in pIMAY between *Sac*I and *Not*I restriction sites. The amplified fragment was designed to recombine with the Sa17’s *recA* gene in the chromosome, resulting in integration of the plasmid. For the amplification of the Sa17 *recA* fragment Phusion polymerase was used and a PCR reaction with initial denaturation at 9 °C for 30 s, a first denaturation, annealing and elongation step for 10 cycles at 98 °C for 10 s, 57 °C for 10 s, 72 °C for 30 s, respectively, a second denaturation, annealing and elongation step for 25 cycles at 98 °C for 10 s, 72 °C for 30 s, respectively, followed by a final elongation at 72 °C for 5 min. The product size was confirmed by gel electrophoresis and purified using GeneJet PCR purification kit (Thermo Fisher Scientific, Waltham, MA, USA). Both the PCR product and pIMAY were digested with *Sac*I and *Not*I and ligated with a molar ratio of 1:3 creating a pIMAY+*recA-*disruption mutant plasmid. Preparation of electro-competent *S. aureus* Sa17 cells and transformation were performed according to the original protocol of Löfblom *et al.* [24] and as adjusted by Monk *et al.* [23].

Mutant selection was performed as described in Monk *et al.* [23]. Disruption of the *recA* gene was confirmed by colony PCR using primer YBRecA-110 and YBRecA-111 (Table 3). The PCR reaction was performed using Phire Hot Start II DNA Polymerase (Thermo Fisher Scientific, Waltham, MA, USA) and the following protocol: a part of colony was transferred in a PCR tube using a 1 μL loop, heated in the microwave at full power (800 W) for 5 min and immediately cooled down on ice. An amount of 50 μL of master mix containing 1 U·μL^−1^ of DNA polymerase, as recommended by the manufacturer was then added to the PCR tube. Colony PCR reaction was performed at initial denaturation of 98 °C for 30 s, denaturation annealing and elongation (35 cycles) at 98 °C for 10 s, 51 °C for 10 s and 72 °C for 20 s, respectively, followed by final elongation at 72 °C for 5 min.

The Sa17 *recA*-disruption mutant and Sa17 wild type strains were investigated for their growth pattern and SEA production under controlled and induced growth conditions, as described in “Bacterial strains and growth conditions”. Both strains were then studied for the amount of RF copies produced, by amplification of the RF target in a conventional PCR reaction. The primers used were the same as described in “RF assay” (Table 2), the DNA Polymerase was Phire Hot Start II (Thermo Fisher Scientific, Waltham, MA, USA). To normalize the reaction, the same amount of plasmid DNA (10 ng) isolated from each time point was used as template. The reaction set up was as follows: initial denaturation at 98 °C for 30 s, denaturation, annealing and elongation steps for 25 cycles at 98 °C for 10 s, 56.5 °C for 10 s and 72 °C for 10 s, respectively, followed by final elongation at 72 °C for 5 min. Assuming the efficiency of PCR in each reaction is the same, an equal volume of 5 μL from each sample along with GeneRuler DNA Ladder Mix (Thermo Fisher Scientific, Waltham, MA, USA) to verify the product size was visualized in gel electrophoresis using Biorad Universal Hood II (Biorad Laboratories Inc., Hercules, CA, USA). Density analysis of the bands was performed using Quantity one 1-D analysis software (Biorad Laboratories Inc., Hercules, CA, USA).

### 4.10. Statistical Analyses

The mean values for RF and *sea* gene levels between the MMC induced and control conditions were compared for statistical significance using unpaired *t*-test function of Microsoft Office Excel (2013, Microsoft, Stockholm, Sweden) at a significance levels of 0.05 (*p* value).

## 5. Conclusions

In light of these various influential mechanisms, the complexity of *sea* gene regulation is unraveled. It could be proposed that *sea* gene transcription is governed by a network of synergistic signals, originating from both the *S. aureus* cell and the relevant *sea*-carrying prophage as a response to the environment. It becomes evident in this study that the SEA-producing strain, the *Siphoviridae* phage and the food environment must be assessed concomitantly for achieving a realistic SEA hazard evaluation with respect to food safety.

## Supplementary Materials

Table S1: *S. aureus* strains used for the sequencing and alignment of the attachment sites of the respective prophages. Table S2: Measurement of density of amplicons of RF from *S. aureus* strains Sa17 wild type and Sa17 Δ*recA* grown in BHI under induced and control conditions. Figure S1a,b: *S. aureus* strain Sa17 grown in BHI under control and MMC induced conditions. Figure S2a,b: *S. aureus* strain Sa48 grown in BHI under control and MMC induced conditions. Figure S3a,b: *S. aureus* strain Sa21 grown in BHI under control and MMC induced conditions. Figure S4a,b: *S. aureus* strain Mu50 grown in BHI under control and MMC induced conditions. Figure S5: *S. aureus* strain Sa51 grown in BHI under control and MMC induced conditions. Figure S6a–c: Sequence alignment of the start and stop region, respectively, of the *sea* gene region of 11 *S. aureus* strains. Supplementary Materials can be accessed at www.mdpi.com/2072-6651/7/12/4889/s1.
