# Supplementary material for: Prophage-Encoded Staphylococcal Enterotoxin A: Regulation of Production in Staphylococcus aureus Strains Representing Different Sea Regions
Source: Toxins (Basel). 2015 Dec 9;7(12):5359–76. doi: 10.3390/toxins7124889 (PMC4690139; doi:10.3390/toxins7124889)
Supplement: Supplementary file 1 [file toxins-07-04889-s001.pdf]

Supplementary Materials: Prophage-Encoded Staphylococcal Enterotoxin A: Regulation of Production in *Staphylococcus aureus* Strains Representing Different *Sea* Regions

Nikoleta Zeaki, Yusak Budi Susilo, Anna Pregiel, Peter Rådström and Jenny Schelin *

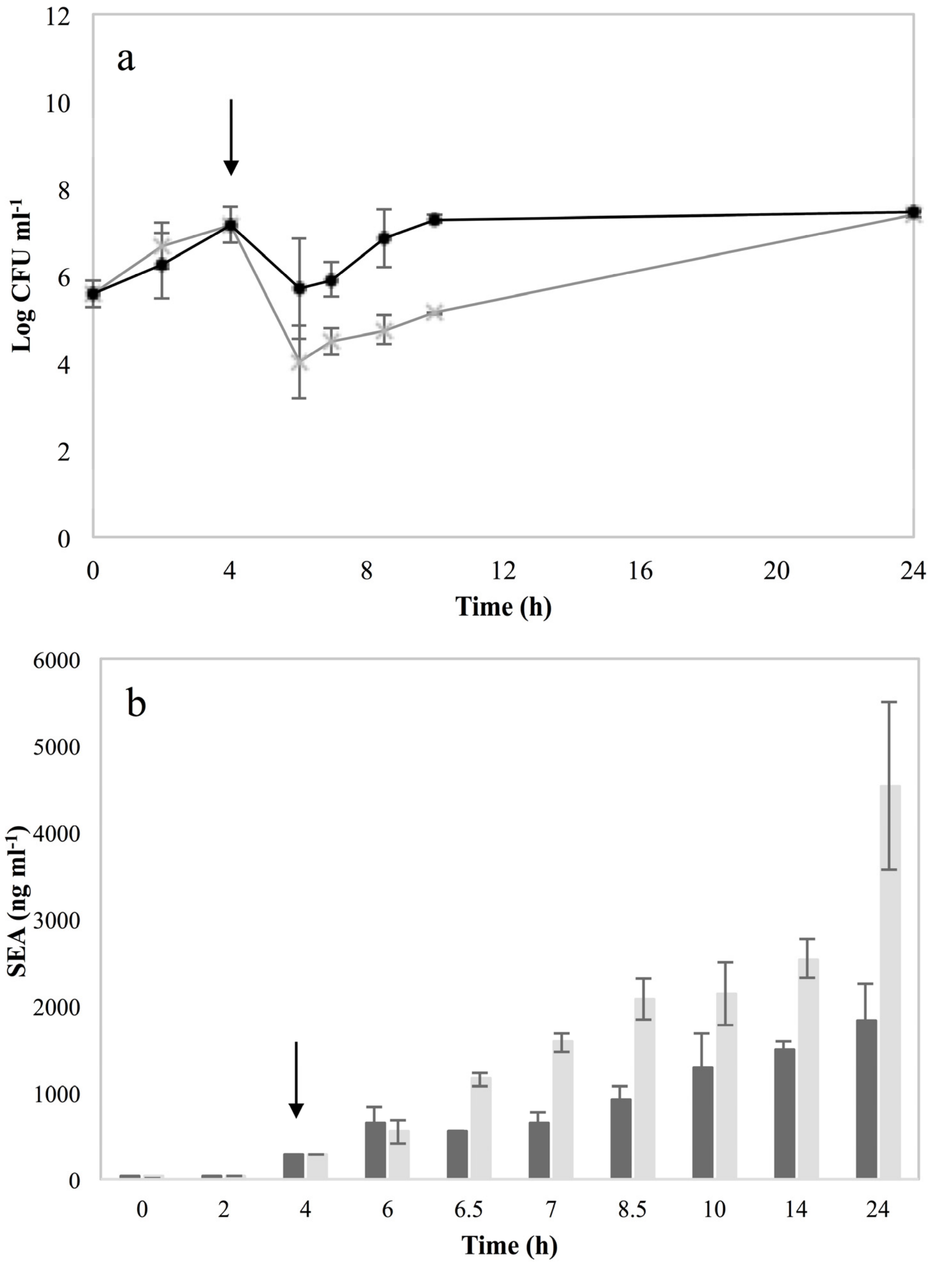

Figure S1. *Staphylococcus aureus* (*S. aureus*) strain Sa17 grown in Brain Heart Infusion (BHI) under control and Mitomycin C (MMC) induced conditions. Average values including standard deviations (⊤, ⊥) of three independent experiments are presented. In MMC induced culture, the point of induction at 4 h is indicated with an arrow. Time is represented in the *X*-axis in hours (h). **Dark grey** bars represent the control culture and **light grey** bars the MMC induced culture. (**a**) Growth in log CFU (*Y*-axis) under control (●) and induced (×) conditions. (**b**) SEA levels (bars) in ng·mL^{-1} of culture (*Y*-axis).

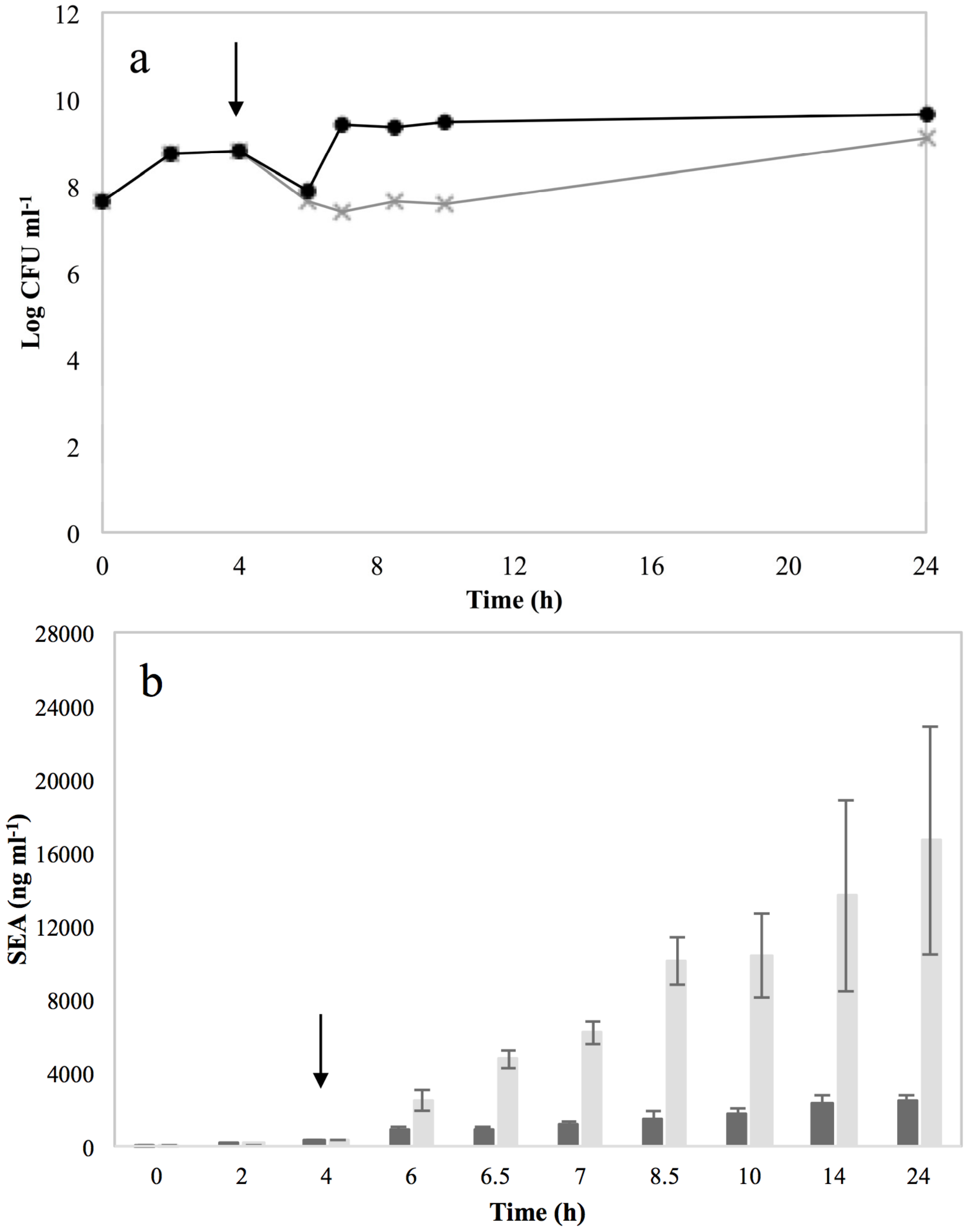

Figure S2. *S. aureus* strain Sa48 grown in BHI under control and MMC induced conditions. Average values including standard deviations (⊤, ⊥) of three independent experiments are presented. In MMC induced culture, the point of induction at 4 h is indicated with an arrow. Time is represented in the *X*-axis in hours (h). **Dark grey** bars represent the control culture and **light grey** bars the MMC induced culture. (**a**) Growth in log CFU (*Y*- axis) under control (●) and induced (×) conditions. (**b**) SEA levels (bars) in ng·mL^{-1} of culture (*Y*-axis).

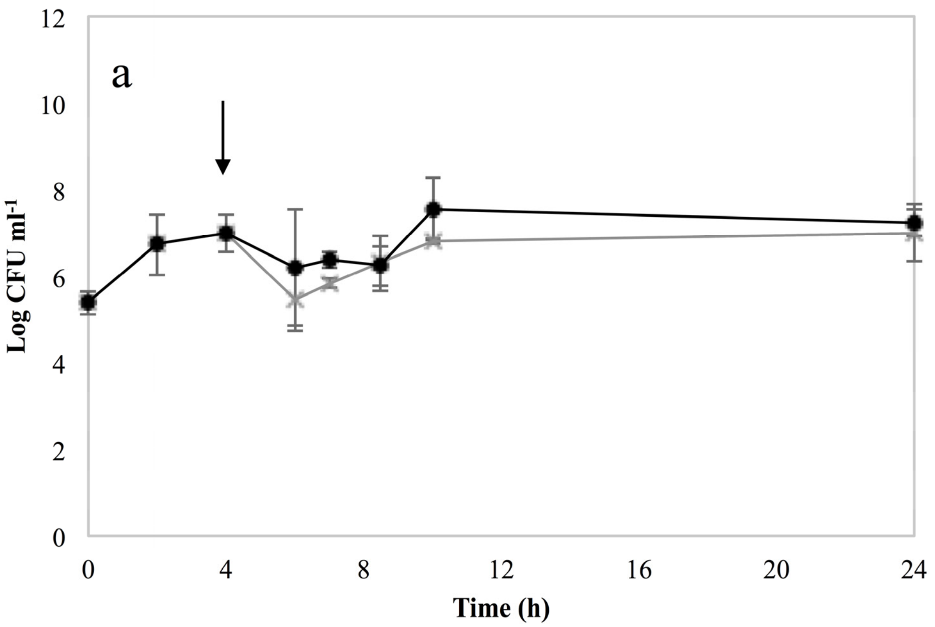

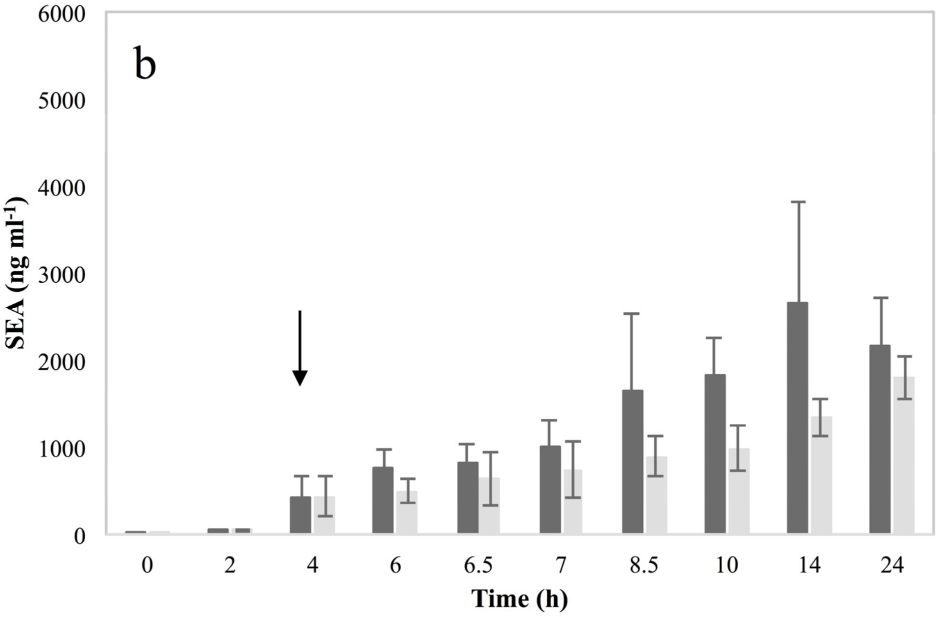

Figure S3. *S. aureus* strain Sa21 grown in BHI under control and MMC induced conditions. Average values including standard deviations (⊤, ⊥) of three independent experiments are presented. In MMC induced culture, the point of induction at 4 h is indicated with an arrow. Time is represented in the *X*-axis in hours (h). **Dark grey** bars represent the control culture and **light grey** bars the MMC induced culture. (**a**) Growth in log CFU (*Y*- axis) under control (●) and induced (×) conditions. (**b**) SEA levels (bars) in ng·mL^{-1} of culture (*Y*-axis).

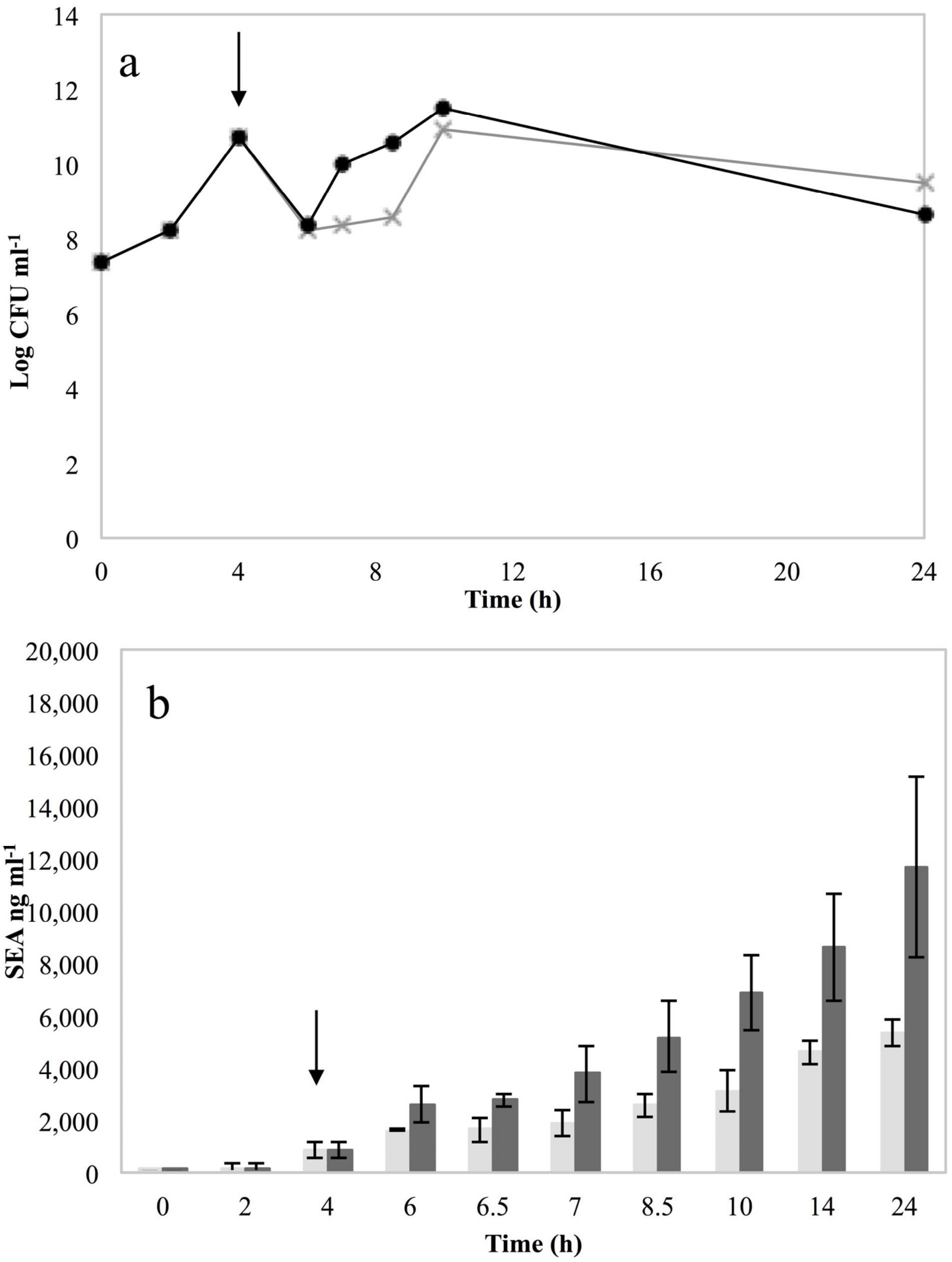

Figure S4. *S. aureus* strain Mu50 grown in BHI under control and MMC induced conditions. Average values including standard deviations (⊤, ⊥) of three technical replicates are presented. In MMC induced culture, the point of induction at 4 h is indicated with an arrow. Time is represented in the *X*-axis in hours (h). **Dark grey** bars represent the control culture and **light grey** bars the MMC induced culture. (**a**) Growth in log CFU (*Y*- axis) under control (●) and induced (×) conditions. (**b**) SEA levels (bars) in ng·mL^{-1} of culture (*Y*-axis).

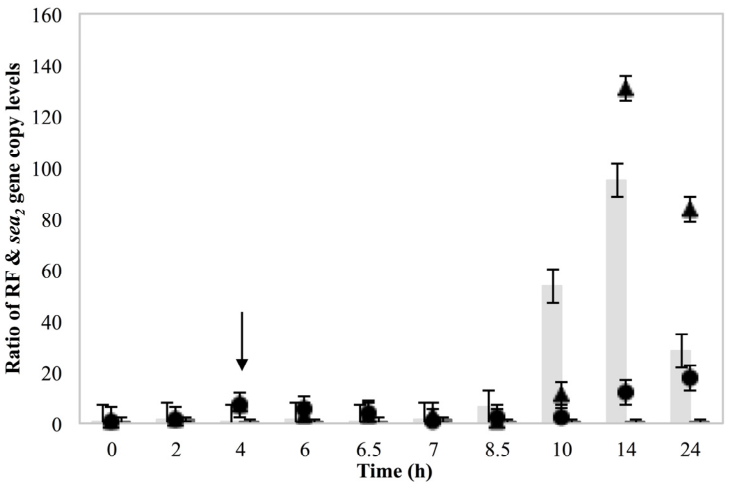

Figure S5. *S. aureus* strain Sa51 grown in BHI under control and MMC induced conditions. Average values including standard deviations (⊤, ⊥) of three technical replicates are presented. In MMC induced culture, the point of induction at 4 h is indicated with an arrow. The *Y*-axis represents the relative ratio of RF (bars) and *sea* gene copy [control (●). induced (▲)] levels while the *X*-axis represents time in hours (h). Dark grey bars represent the control culture and light grey bars the MMC induced culture.

a

```
Sa51    1   TGTCAATACCAAGTAAGTAA GATATCTGAAATGTATAATA GAGTAAAAATAAAATCTTTT TATTATATTATAGACAAGTA
MRSA252 1   TGTCAATACCAAGTAAGTAA GATATCTGAAATGTATAATA GAGTAAAAATGAAATCTTTT TATTATATTATAGACAAGTA
Newman  1   TGTCAATACCAAGTAAGTAA GATATCTGAAATGTATAATA GAGTAAAAATGAAATCTTTT TATTATATTATAGACAAGTA
Sa53    1   TGTTAATACCAAGTAAGTAA GATATCTGAAATGTATAATA GAGTAAAAATGAAATCTTTT -----TATTATAGACAAATA
Sa48    1   TGTTAATACCAAGTAAGTAA GATATCTGAAATGTATAATA GAGTAAAAATGAAATCTTTT -----TATTATAGACAAATA
Sa45    1   TGTTAATACCAAGTAAGTAA GATATCTGAAATGTATAATA GAGTAAAAATGAAATCTTTT -----TATTATAGACAAATA
Sa17    1   TGTTAATACCAAGTAAGTAA GATATCTGAAATGTATAATA GAGTAAAAATGAAATCTTTT -----TATTATAGACAAATA
Sa54    1   TGTTAATACCAAGTAAGTAA GATATCTGAAATGTATAATA GAGTAAAAATGAAATCTTTT -----TATTATAGACAAATA
Sa21    1   TGTTAATACCAAGTAAGTAA GATATCTGAAATGTATAATA GAGTAAAAATGAAATCTTTT -----TATTATAGACAAATA
MW2     1   TGTTAATACCAAGTAAGTAA GATATCTGAAATGTATAATA GAGTAAAAATGAAATCTTTT -----TATTATAGACAAATA
Mu50    1   TGTTAATACCAAGTAAGTAA GATATCTGAAATGTATAATA GAGTAAAAATGAAATCTTTT -----TATTATAGACAAATA
            *** **************** ******************** ********** *********      ************ **

Sa51    81  TAAAAAAGGTATAGTAATAT ATGTATGTATAAGTAAATAA TGATCATTCCATAATTATTG TATATAACT-AATAATTACT
MRSA252 81  TAAAAAAGGTATAGTAATAT ATGTATGTATAAGTAAATAA TGATAATTCTATAATTATTG TATATAACT-AATAATTACT
Newman  81  TAAAAAAGGTATAGTAATAT ATGTATGTATAAGTAAATAA TGATAATTCTATAATTATTG TATATAACT-AATAATTACT
Sa53    81  TAAAAAGTGTATAGTAATAT ATGTATGTATAATTAAATGA TAATCATTTCATAATTATTG TATATAACTAAATAACTACT
Sa48    81  TAAAAAGTGTATAGTAATAT ATGTATGTATAATTAAATGA TAATCATTTCATAATTATTG TATATAACTAAATAACTACT
Sa45    81  TAAAAAGTGTATAGTAATAT ATGTATGTATAATTAAATGA TAATCATTTCATAATTATTG TATATAACTAAATAACTACT
Sa17    81  TAAAAAGTGTATAGTAATAT ATGTATGTATAATTAAATGA TAATCATTTCATAATTATTG TATATAACTAAATAACTACT
Sa54    81  TAAAAAGTGTATAGTAATAT ATGTATGTATAATTAAATGA TAATCATTTCATAATTATTG TATATAACTAAATAACTACT
Sa54    81  TAAAAAGTGTATAGTAATAT ATGTATGTATAATTAAATGA TAATCATTTCATAATTATTG TATATAACTAAATAACTACT
MW2     81  TAAAAAGTGTATAGTAATAT ATGTATGTATAATTAAATGA TAATCATTTCATAATTATTG TATATAACTAAATAACTACT
Mu50    81  TAAAAAGTGTATAGTAATAT ATGTATGTATAATTAAATGA TAATCATTTCATAATTATTG TATATAACTAAATAACTACT
            ******  ************ ************ ***** * * ** ***  ********** ********* ***** ****
                                                                                          START
Sa51    161 CCAACAAAAATAATCTATTA TACAAAAATTTCGGATAATA ACAAGTTTATATGGAATTAT GCTTTAGAGGTGAGTAAAAT
MRSA252 161 TCGACAAAAATAATCTATTA TCCAAATATTTTAGATAATA AAAAGTTTGTATGGAATTAT GCTTTAGAGGTGAGCAAAAT
Newman  161 TCGACAAAAATAATCTATTA TCCAAATATTTTAGATAATA AAAAGTTTGTATGGAATTAT GCTTTAGAGGTGAGCAAAAT
Sa53    161 T-AACAAAAAT--------- -------------------- --------------AATTAT GCTTTAGAGGTGAGCAAAAT
Sa48    161 T-AACAAAAAT--------- -------------------- --------------AATTAT GCTTTAGAGGTGAGCAAAAT
Sa45    161 T-AACAAAAAT--------- -------------------- --------------AATTAT GCTTTAGAGGTGAGCAAAAT
Sa17    161 T-AACAAAAAT--------- -------------------- --------------AATTAT GCTTTAGAGGTGAGCAAAAT
Sa54    161 T-AACAAAAAT--------- -------------------- --------------AATTAT GCTTTAGAGGTGAGCAAAAT
Sa21    161 T-AACAAAAAT--------- -------------------- --------------AATTAT GCTTTAGAGGTGAGCAAAAT
MW2     161 T-AACAAAAAT--------- -------------------- --------------AATTAT GCTTTAGAGGTGAGCAAAAT
Mu50    161 T-AACAAAAAT--------- -------------------- --------------AATTAT GCTTTAGAGGTGAGCAAAAT
               ********                                            ****** ************** *****

Sa51    241 AAAAAAAACAACATTTATAC TACTTTCATTTATTGCCCTA ACGTTGATAACAAGTCCATT
MRSA252 241 GAAAAAAACAGCATTTATAC TACTTTTATTCATTGCCCTA ACGTGGACAACAAGTCCACT
Newman  241 GAAAAAAACAGCATTTATAC TACTTTTATTCATTGCCCTA ACGTGGACAACAAGTCCACT
Sa53    241 GAAAAAAACAGCATTTACAT TACTTTTATTCATTGCCCTA ACGTTGACAACAAGTCCACT
Sa48    241 GAAAAAAACAGCATTTACAT TACTTTTATTCATTGCCCTA ACGTTGACAACAAGTCCACT
Sa45    241 GAAAAAAACAGCATTTACAT TACTTTTATTCATTGCCCTA ACGTTGACAACAAGTCCACT
Sa17    241 GAAAAAAACAGCATTTACAT TACTTTTATTCATTGCCCTA ACGTTGACAACAAGTCCACT
Sa54    241 GAAAAAAACAGCATTTACAT TACTTTTATTCATTGCCCTA ACGTTGACAACAAGTCCACT
Sa21    241 GAAAAAAACAGCATTTACAT TACTTTTATTCATTGCCCTA ACGTTGACAACAAGTCCACT
MW2     241 GAAAAAAACAGCATTTACAT TACTTTTATTCATTGCCCTA ACGTTGACAACAAGTCCACT
Mu50    241 GAAAAAAACAGCATTTACAT TACTTTTATTCATTGCCCTA ACGTTGACAACAAGTCCACT
             ********* ****** *  ****** *** ********* **** ** ********** *
```

Figure S6. *Cont.*

b

```
Sa51    1   TTTATTTGGTGCTCAAGGAC AATATCCAGATACACAGTTG AGGATATATAGAGATAATAA AACGATTAAGTCTAAAAATA
MRSA252 1   TTTATTTGGTGCTCAAGGAC AGAATTCAAATACACTATTA AGAATATATAGAGATAATAA AACGATTAACTCTGAAAACA
Newman  1   TTTATTTGGTGCTCAAGGAC AGAATTCAAATACACTATTA AGAATATATAGAGATAATAA AACGATTAACTCTGAAAACA
Sa53    1   TTTATTTGGTGCTCAAGGAC AGTATTCAAATACACTATTA AGAATATATAGAGATAATAA AACGATTAACTCTGAAAACA
Sa48    1   TTTATTTGGTGCTCAAGGAC AGTATTCAAATACACTATTA AGAATATATAGAGATAATAA AACGATTAACTCTGAAAACA
Sa45    1   TTTATTTGGTGCTCAAGGAC AGTATTCAAATACACTATTA AGAATATATAGAGATAATAA AACGATTAACTCTGAAAACA
Sa17    1   TTTATTTGGTGCTCAAGGAC AGTATTCAAATACACTATTA AGAATATATAGAGATAATAA AACGATTAACTCTGAAAACA
Sa54    1   TTTATTTGGTGCTCAAGGAC AGTATTCAAATACACTATTA AGAATATATAGAGATAATAA AACGATTAACTCTGAAAACA
Sa21    1   TTTATTTGGTGCTCAAGGAC AGTATTCAAATACACTATTA AGAATATATAGAGATAATAA AACGATTAACTCTGAAAACA
MW2     1   TTTATTTGGTGCTCAAGGAC AGTATTCAAATACACTATTA AGAATATATAGAGATAATAA AACGATTAACTCTGAAAACA
Mu50    1   TTTATTTGGTGCTCAAGGAC AGTATTCAAATACACTATTA AGAATATATAGAGATAATAA AACGATTAACTCTGAAAACA
            ******************** *  ** ** ******  **  ** ***************** ********* *** **** *

                                         STOP
Sa51    81  TGCATATTGATATATATTTA TATACAACTTGAGTGAAATT TTGGCTGAAAAAATTTAAAA TCTATAAAAAGTACCATAAG
MRSA252 81  TGCATATTGATATATATTTA TATACAAGTT---------- -------------------- --------------------
Newman  81  TGCATATTGATATATATTTA TATACAAGTT---------- -------------------- --------------------
Sa53    81  TGCATATTGATATATATTTA TATACAAGTT---------- -------------------- --------------------
Sa48    81  TGCATATTGATATATATTTA TATACAAGTT---------- -------------------- --------------------
Sa45    81  TGCATATTGATATATATTTA TATACAAGTT---------- -------------------- --------------------
Sa17    81  TGCATATTGATATATATTTA TATACAAGTT---------- -------------------- --------------------
Sa54    81  TGCATATTGATATATATTTA TATACAAGTT---------- -------------------- --------------------
Sa21    81  TGCATATTGATATATATTTA TATACAAGTT---------- -------------------- --------------------
MW2     81  TGCATATTGATATATATTTA TATACAAGTT---------- -------------------- --------------------
Mu50    81  TGCATATTGATATATATTTA TATACAAGTT---------- -------------------- --------------------
            ******************** ******* **

Sa51    161 TTATTTTATTACTATACTTA AAACATTCACTTTTTTACTG CTGTTTGTAAGTTTTATAAA AAATTTCTATAGATAGTTTT
MRSA252 161 -------------------- AAACATGGTAGTTTTGAACA C-----GTAATGTT------ ---------CAGATTATTAT
Newman  161 -------------------- AAACATGGTAGTTTTGAACA C-----GTAATGTT------ ---------CAGATTATTAT
Sa53    161 -------------------- AAACATGGTAGTTTTGACCA C-----GTAATGTT------ ---------CAGATTATTAT
Sa48    161 -------------------- AAACATGGTAGTTTTGACCA C-----GTAATGTT------ ---------CAGATTATTAT
Sa45    161 -------------------- AAACATGGTAGTTTTGACCA C-----GTAATGTT------ ---------CAGATTATTAT
Sa17    161 -------------------- AAACATGGTAGTTTTGACCA C-----GTAATGTT------ ---------CAGATTATTAT
Sa54    161 -------------------- AAACATGGTAGTTTTGACCA C-----GTAATGTT------ ---------CAGATTATTAT
Sa21    161 -------------------- AAACATGGTAGTTTTGACCA C-----GTAATGTT------ ---------CAGATTATTAT
MW2     161 -------------------- AAACATGGTAGTTTTGACCA C-----GTAATGTT------ ---------CAGATTATTAT
Mu50    161 -------------------- AAACATGGTAGTTTTGACCA C-----GTAATGTT------ ---------CAGATTATTAT
                                 ******     ****  *   *     ****  **                ****  ** *

Sa51    241 GAACAAAGCGAGAATAATCT GAAAGTATACAAGCAGTAAA AAAAGTATATGTGCTATAAT
MRSA252 241 GAAC----CGAGAATAATCT GAAAGTTTACAAGCAGTAAA AAAAGTATATGTGCTATAAT
Newman  241 GAAC----CGAGAATAATCT GAAAGTTTACAAGCAGTAAA AAAAGTATATGTGCTATAAT
Sa53    241 GAAC----CGAGAATAATCT AAAAGTTTACAAGCAGTAAA AAAAGTATATGTGCTATAAT
Sa48    241 GAAC----CGAGAATAATCT AAAAGTTTACAAGCAGTAAA AAAAGTATATGTGCTATAAT
Sa45    241 GAAC----CGAGAATAATCT AAAAGTTTACAAGCAGTAAA AAAAGTATATGTGCTATAAT
Sa17    241 GAAC----CGAGAATAATCT AAAAGTTTACAAGCAGTAAA AAAAGTATATGTGCTATAAT
Sa54    241 GAAC----CGAGAATAATCT AAAAGTTTACAAGCAGTAAA AAAAGTATATGTGCTATAAT
Sa21    241 GAAC----CGAGAATAATCT AAAAGTTTACAAGCAGTAAA AAAAGTATATGTGCTATAAT
MW2     241 GAAC----CGAGAATAATCT AAAAGTTTACAAGCAGTAAA AAAAGTATATGTGCTATAAT
Mu50    241 GAAC----CGAGAATAATCT AAAAGTTTACAAGCAGTAAA AAAAGTATATGTGCTATAAT
            ****    ************  ***** ************* ********************
```

c

```
MW2,Sa17 SEA   1     MKKTAFTLLLFIALTLTTSPLVNGSEKSEEINEKDLRKKSELQGTALGNLKQIYYYNEKA
                    #|||#|#||:||||||#|||:||#||||||||:|||#|||||||||||:||:|:||:|::|
Sa51 SEA       1     IKKTTFILLSFIALTLITSPFVNCSEKSEEINGKDLQKKSELQGTALSNLRQTYYHNGSA

MW2,Sa17 SEA   61    KTENKESHDQFLQHTILFKGFFTDHSWYNDLLVDFDSKDIVDKYKGKKVDLYGAYYGYQC
                    ##|||||:||||::||||::|||:|#||||||||::|||:::#||||||||||:||||||
Sa51 SEA       61    IIENKESNDQFLKNTILFNDFFTGHQWYNDLLVDLGSKDTANIYKGKKVDLYGVYYGYQC

MW2,Sa17 SEA   121   AGGTPNKTACMYGGVTLHDNNRLTEEKKVPINLWLDGKQNTVPLETVKTNKKNVTVQELD
                    #||||#|||||||||||||||#|#||||||||||:||||||||||:|||||||#|||||||
Sa51 SEA       121   TGGTPFKTACMYGGVTLHDNNQLEEEKKVPINLWIDGKQNTVPLGTVKTNKKEVTVQELD

MW2,Sa17 SEA   181   LQARRYLQEKYNLYNSDVFDGKVQRGLIVFHTSTEPSVNYDLFGAQGQYSNTLLRIYRDN
                    ||#::||:|:|||||#|:|:||#|||||#||#|#::||:|||||||||||#:|:|||||||
Sa51 SEA       181   LQSKHYLHETYNLYNTDAFNGKIQRGLIEFHPSSGDSVGYDLFGAQGQYPDTQLRIYRDN

MW2,Sa17 SEA   241   KTINSENMHIDIYLYTS
                    |||:|:||||||||||#
Sa51 SEA       241   KTIKSKNMHIDIYLYTT
```

Figure S6. (**a**,**b**) Sequence alignment of the start and stop region, respectively, of the *sea* gene region of 11 *S. aureus* strains. Start and stop codons are indicated with boxes. Differences in the sequences are indicated by the absence of star sign under the sequences. (**c**) Alignments of *S. aureus* MW2, Sa17 and Sa51 SEA amino acid sequences. Differences (59 amino acids) are highlighted in grey. The colon symbol (:) denotes a conservative mutation and the hash symbol (#) is denoting a non-conservation mutation.

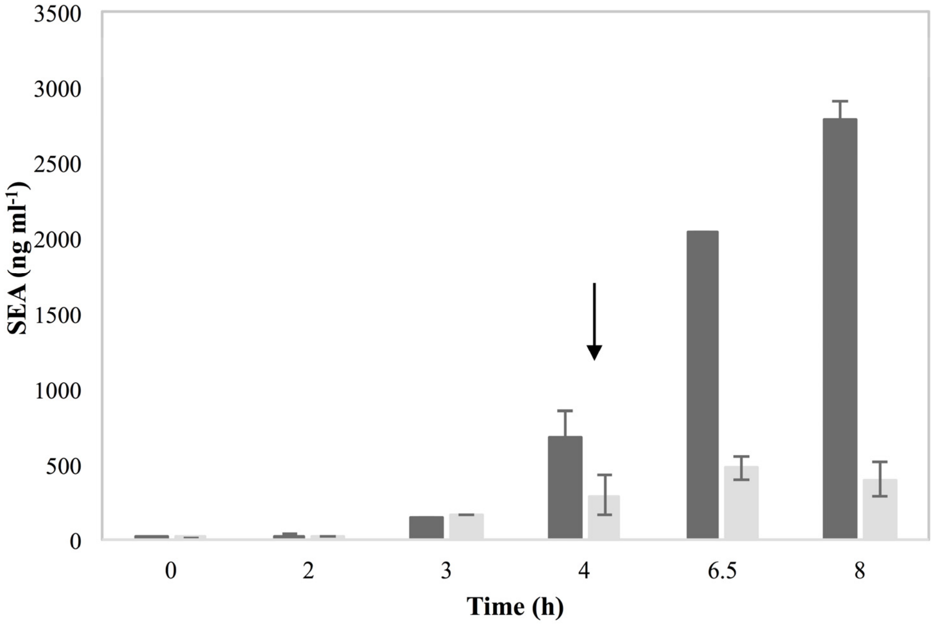

Figure S7. *S. aureus* strain Sa17 *recA*-disruption mutant grown in BHI under control and MMC induced conditions. Average values including standard deviations (⊤, ⊥) of three technical replicates are presented. In MMC induced culture, the point of induction at 4 h is indicated with an arrow. Time is represented in the *X*-axis in hours (h). The Y axis represents SEA levels in ng·mL^{-1} of culture in **Dark grey** bars for the control culture and **light grey** bars for the MMC induced culture.

Table S1. *S. aureus* strains used for the sequencing and alignment of the attachment sites of the respective prophages.

Strain Name	NCBI Accession Number
**Mu50**	KT253887
**Sa17**	KT253888
**Sa48**	KT253889
**Sa51**	KT253890
**Sa54**	KT253891

Table S2. Measurement of density of amplicons of RF from *S. aureus* strains Sa17 wild type and Sa17 Δ*recA* grown in BHI under induced and control conditions.

Sample	Mean Value Intensity (INT)
**2 h_a**	220.22
**2 h_b**	215.40
**3 h_a**	323.28
**3 h_b**	354.14
**4 h_a**	540.90
**4 h_b**	454.30
**6.5 h_a**	526.96
**6.5 h_b**	865.06
**8 h_a**	386.51
**8 h_b**	931.23
**pos. control**	1284.69
**neg. control**	214.12
